# Analysis of complications in hepatic right lobe living donors

**DOI:** 10.4103/0256-4947.59368

**Published:** 2010

**Authors:** Ayman Azzam, Kinji Uryuhara, Ito Taka, Yasutsugu Takada, Hiroto Egawa, Koichi Tanaka

**Affiliations:** a From the Department of General Surgery, University of Alexandria, Egypt, Japan; b From the Department of Transplantation and Immunology, Kyoto University, Kyoto, Japan; c From the Institute of Biochemical Research and Innovation, Kobe, Japan

## Abstract

**BACKGROUND AND OBJECTIVES:**

Living donor liver transplantation (LDLT) has been expanding to adult recipients by using right lobe grafts. However, the incidence of complications is more frequent than that involving left lobe grafts. Hence, we aimed to analyze postoperative complications in right lobe liver donors as a step to improve the results in the donors.

**METHODS:**

Three hundred and eleven right lobe liver donors were retrospectively reviewed between February 1998 and December 2003.

**RESULTS:**

The ages of the donors ranged from 19 to 64 years (median: 46 years). Their body mass index ranged from 16.6 to 34.3 (median: 22.1). The mean duration of the operation was 6.58 (1.25) hours and blood loss was 289 (254) mL. The estimated median donor residual liver volume was 42.2% (range: 20.6-60.3%) and the median hospital stay was 14.5 days (range: 6-267 days). One donor died of liver failure due to small residual liver volume (26%) and steatohepatitis. One hundred and twenty three complications occurred in 104 donors (33.4%). Donors experienced one or more complications. According to the Clavien classification, grade I complications occurred in 71 of the episodes (57.7%), grade II complications in 9 (7.3%), grade IIIa complications in 39 (31.7%), grade IIIb complications in 3 (2.5%), and grade V complications in 1 (0.8%). Biliary complications were the most common and occurred in 37 donors (12%).

**CONCLUSION:**

Right lobe liver donation is a widely accepted procedure that results in the expansion of the indication for LDLT to adults and large children. However, remnant liver size and anatomical variations in the biliary tree represent important risk factors for postoperative complications.

The shortage of donor organs for liver transplantation has led to interest in alternatives to cadaver liver transplantation. Living donor liver transplantation (LDLT), using the left lateral segment or the left lobe, is an acceptable modality to treat end-stage liver disease, especially in pediatric patients. This procedure provides adequate graft mass with excellent results and better donor safety in pediatric patients.^1^ However, small-for-size grafts has remained a significant barrier with high morbidity after the expansion of LDLT to adult patients.^2^,^3^ Recently, right hepatic lobe donation had been advocated to overcome size-mismatch between the left lobe and a large-sized LDLT recipient.^4^–^5^ Right lobe donation usually matches graft size although donor safety remains a major concern. In general, a normal liver has been reported to be able to tolerate right lobectomy well, which results in a residual liver volume (RLV) that is as small as 20 to 40%.^6^–^8^ However, surgery-related morbidity, safety of right-lobe donors, and postoperative donor liver function have to be fully investigated and re-evaluated. As donor safety is the top priority in LDLT, the present study focused on the analysis of postoperative complications in right-lobe donors.

## METHODS

### Study population

Donor candidates were limited to relatives up to the third degree and spouses who showed a strong voluntary wish to donate parts of their livers. After the first-step of obtaining informed consent, donor candidates were subjected to medical screening consisting of blood chemistry analysis, blood count, check for viral hepatitis, abdominal ultrasonography (US), and other tests for general anesthesia. The final candidate was subjected to liver volumetry by using computed tomography (CT). Magnetic resonance cholangiography was performed in a limited number of donors (n=26) with variations in portal venous anatomy such as trifurcation.

Selection criteria for donors were age between 20 and 60 years, healthy, ABO-compatible, graft size >1.0% of recipient body weight, and residual liver volume (RLV=estimated whole liver volume - actual graft weight)/estimated whole liver volume ×100) >30% of the whole liver. Medical or surgical risk factors disclosed by donors were investigated further, if indicated, and final candidacy was determined by a multidisciplinary team. Exceptions in each factor were accepted due to the scarcity of available donors and the severity of the disease in the recipients.

Whole-liver volume and estimated graft and RLV in donors were calculated preoperatively on radiographic CT. CT of the liver for volume determination was performed as described elsewhere.^9^,^10^ No donor candidate was subjected to angiography for arterial anatomy or endoscopic retrograde cholangiography for biliary anatomy. Steatosis of the liver was evaluated only on US and CT.^11^ Hepatic steatosis was detected by CT by measuring the liver-to-spleen (L/S) CT value ratio. Steatosis was suspected when the L/S ratio is ≤1.1.^11^,^12^ Such donors were advised to receive dietary and exercise treatment and to abstain from alcohol before donation and reevaluated later. No liver biopsy was performed preoperatively, as per protocol.

### Donor operation

Donor operations were performed as described elsewhere:^13^ first, complete mobilization of the right lobe, followed by transection of the short hepatic veins on the right side of the inferior vena cava, except in the case of significantly sized right inferior hepatic vein (5 mm). This was followed by isolation of the right branches of the portal vein, hepatic artery, and hepatic duct. At this stage, intraoperative cholangiography was usually performed through the cystic duct and the right hepatic duct(s) was usually transected before the parenchymal transection was started. Transection of the liver was subsequently performed. The demarcation line for transection was determined by temporary clamping of the right portal pedicle. After graft extraction, it was flushed *ex situ* with a histidine-tryptophan-ketoglutarate (HTK) solution through the right branch(es) of the portal vein, weighed, and preserved in the same solution until the time of graft implantation.

### Postoperative management

Postoperative complications were recorded during and after the initial hospital stay. The follow-up period for the donors was 11 to 81 (median: 32) months. Clavien classification^14^ was used to record surgical complications as follows: grade I, alterations from the ideal postoperative course not requiring specialized pharmacological or surgical treatment; grade II, complications requiring specialized pharmacological treatment, blood transfusion, or total parental nutrition; grade III-a, complications requiring invasive intervention without general anesthesia; grade III-b, requiring general anesthesia; grade IV-a, single organ dysfunction; grade IV-b, multiorgan dysfunction; grade V, death; the suffix “d” indicated disability.

Statistical calculations for mean values and standard deviations were performed using the SPSS software package. The chi-square test was adopted for statistical comparison and *P* <.05 were regarded as being statistically significant.

## RESULTS

In the period between February 1998 and December 2003, 311 liver transplantations were performed using the right lobe from living donors in the Department of Transplantation and Immunology, Kyoto University, Kyoto, Japan. There were 284 right lobe grafts without the middle hepatic vein (MHV) and 27 with the MHV. Donor demographics are shown in Table 1. The graft donors consisted of 162 men and 149 women and their age ranged from 19 to 64 (median: 46) years. The body weight of the donors ranged from 38.4 to 107.2 (median: 61.16) kg and the body mass index (BMI) from 16.6 to 34.3 (median 22.1) kg/m^2^. The donors consisted of 32 fathers, 48 mothers, 78 spouses (33 husbands and 45 wives), 73 siblings (40 brothers and 33 sisters), 73 children (51 sons and 22 daughters), one uncle, one aunt, three nephews, one cousin, and one mother-in-law of the recipients. Thirty-seven donors were ABO-incompatible with their recipients and these ABO-incompatible grafts were selected because of the absence of other compatible candidates. Significant medical history included hypertension in 21 donors, gastro-duodenal ulcer in eight, cardiac arrhythmia in six, diabetes mellitus in seven, anemia in five, asthma in four, angina pectoris in two, gout in two, hyperlipidemia in two, esophageal hernia in one, acute pancreatitis in one, tuberculosis (treated) in one, pyelonephritis in two, pneumonia in one, leiomyoma and hypermennorrhea in two, Kawasaki's disease in one, meningitis in one, multiple liver cysts and solid hemangioma one donor. Previous abdominal surgeries appendicectomy in 40 donors, gynecological surgery in 20, gasterectomy in 1, cholecystectomy in 1, open abdominal biopsy in 1, lumbar disc hernia in 1, cleft lip in 1, nasal fracture operation in 1, mastectomy for cancer breast in 1, and tonsillectomy in one donor. The donors also included 14 positive for hepatitis B surface antigen and two positive for hepatitis C virus (HCV) antibody (status postinterferon therapy), who were adopted as donors in emergency situations as life-saving procedures for recipients positive for the same hepatitis virus.

**Table 1:** Donor demographics.

Sex (M/F)	162/149
Age (years)	19-64 (46)
Body weight (kg)	38.4-107.2 (61.2)
BMI (kg/m^2^)	16.6-34.3 (22.1)

Numbers in parentheses are median values; BMI, body mass index; HBcAb, hepatitis B core antibody

### Surgery and graft profiles

Surgery and graft profiles are shown in Table 2. No allotransfusion was needed for any of the donors during the operation. Residual liver volume (RLV, %) ranged from 20.6% to 60.2% (median 42%). Alternatively, estimated RLV (percent), which uses estimated graft volume instead of actual graft volume, ranged from 20.7% to 55.8% (median 41.2%). Ten donors had a preoperative estimate RLV of less than 30%.

**Table 2:** Surgical and graft profile.

Surgical time (hr)	3.25-11.15 (6.6)
Blood loss (mL)	10-2300 (282)
Graft weight (g)	415-1270 (670.95)
GRWR (%)	0.55-2.39 (1.16)
Estimated RLV (% total)	20.7-55.82 (41.2)
Residual liver volume (% total)	20.6-60.2 (42.0)
Macrovesicual steatosis (mild to moderate)	20.0%

Numbers in parentheses are median values; GRWR, graft-to-recipient weight ratio; RLV, residual liver volume

As the safety limit was tentatively estimated as 30% for the estimated RLV, the actual transection line was intentionally shifted to the right in these ten cases. Consequently, only five of these ten donors had a postoperative estimated-actual RLV<30%. On the other hand, nine other donors had an estimated RLV<30% and had a postoperative estimated-actual RLV>30%. It was found that the rate of postoperative complications was significantly higher in donors with postoperative estimated-actual RLV<30% with *P*=.001.

### Postoperative liver functions

Aspartate aminotransferase (AST), alanine aminotransferase (ALT), and serum total bilirubin (TB) levels returned to normal levels after the end of the first week in 15% of the donors. These levels had returned to normal in 50% and 90% of the donors by the end of the second and third weeks respectively. AST, ALT, and serum total bilirubin had decreased to normal levels within one month after the transplant surgery in all donors. The mean peak levels for AST, ALT, and serum TB were 348.8 (151.7) U/L, 354 (174.9) U/L, and 3.2 (1.4) mg/dL respectively. On the seventh postoperative day, AST, ALT, and TB levels were 69.6 (35.8 U/L), 128 (62.4 U/L), and 1.28 (1.04 mg/dL). The hospital stay was for 14.5 days (range: 6-267 days).

### Postoperative complications

All (but one) donors are alive and healthy along the follow up period (which extends to 7 years), although 30% of the donors experienced one or more complications. One donor died of liver failure due to a small remnant liver (26%) and concomitant nonalcoholic steatohepatitis which was diagnosed postoperatively in spite of a rescue domino transplant. The preoperative ultrasound and CT showed mild to moderate steatosis and volumetry showed the residual volume to be 30%. The steatohepatitis was diagnosed by liver biopsy taken during the operation. After the operation, the donor developed continuous progressive liver failure which ended with a domino liver transplantation. After initial improvement, the condition deteriorated again until death due to multiple organ failure and sepsis after 267 days of hospital stay. As any complication in a healthy donor is significant, both minor and major complications were recorded and consequently, a total of 123 complications were recorded to occur in 104 donors (Table 3).

**Table 3:** Postoperative complications.

	Number of episodes	Clavien classification				
		GI	GII	GIIIa	GIIIb	GV
Biliary complications						
Bile leakage	34	8		26		
Biliary stricture	6^a^^b^			5	1	
Other abdominal complications						
Persistent fluid collection	38	38				
Skin wound infection	11	11				
Massive ascites (>500 mL/day)	4			4		
Small bowel obstruction	5^b^		4		1	
Hyperamylasemia (>300 IU/L)	3	3				
Portal vein thrombosis	1b				1	
Drug induced hepatotoxicity	1		1			
Diverticulitis (ascending colon)	1		1			
Allergic reaction	1	1				
Extra-abdominal complications						
Pleural effusion	4			4		
Pulmonary embolism (including suspected cases)	3		3			
Fever of unknown origin	8	8				
Angina pectoris	1	1				
Radial nerve palsy (temporary)	1	1				
Death	1					1

**Total**	**123**	**71**	**9**	**39**	**3**	**1**

a Three episodes followed bile leak

b One each case required relaparotomy

Biliary complications were the most common complications and constituted 40/122 episodes (32.7%) in 37/311 donors (about 12 % of all donors); 34/40 bile leakages (85% of biliary complications) and 6/40 biliary strictures (15% of biliary complications) (three of which followed biliary leakage). Biliary leakage was diagnosed with a laboratory test for bilirubin in the drain in 22 cases, hepatobiliary imino-diacetic acid (HIDA) scan in ten cases, and endoscopic retrograde cholangiopancreatography (ERCP) in five cases. The cause of the leakage was the cut surface of the liver in 30 donors (81%) and the site of cut of the bile duct in three donors (8%) but not defined in four donors (11%). All biliary leakages were managed conservatively, either by drainage through the drainage tube until spontaneous disappearance in eight cases, or additional percautaneous drainage after the removal of the drain tube in 15 cases, or the receipt of endoscopic nasobiliary drainage after percautaneous drainage in 12 cases. Bile leakage occurred between one and 41 (median 13) days after donation. Six biliary strictures occurred, three of them followed bile leakage. They were diagnosed on the basis of elevated total serum bilirubin levels and by ERCP. One patient required conversion to hepaticojejunostomy 20 months after donation. The remaining cases were managed successfully with temporary endoscopic retrograde biliary balloon dilatation and stenting. During the period of follow-up, the stent was changed after six months in four donors with biliary strictures and was removed from the other two donors without any complication. Cholestasis occurred in 17 donors (5%) when the total bilirubin levels reached >5 mg/dL. These donors improved upon the administration of urthodesoxycholic acid medication. Biliary ducts were one in 249 donors (80%), two ducts in 59 donors (19%), and three ducts in three donors (1%). The biliary complications due to bile duct leakages (not in the cut surface) were significantly more in the donors with more than one bile duct (*P*=.0001).

Other abdominal complications include persistent asymptomatic fluid collection diagnosed by ultrasonography without any symptoms in 38 donors (12%). Fluid collection was not related to any known risk factors for donors such as steatosis, medical problems, or residual volume. One of the five cases had small bowel obstruction and required surgical adhesiolysis ten months after donation.

Portal vein thrombosis occurred in one patient which was diagnosed on day 7 by the sudden appearance of ascites along with a loss of the portal flow signal on Doppler US. Emergency laparotomy revealed a kink in the reconstructed portal vein. This area was dissected from the surrounding tissue and the kink was corrected. No further problems were encountered with portal vein flow in the follow-up period.

Pleural effusion necessitating drainage occurred in four cases and pulmonary embolism was suspected in three cases with mild hypoxemia and perfusion defects in pulmonary scintigrams. All the three cases recovered with the use of heparin and urokinase.

According to Clavien classification,^14^ there were 123 complications which occurred in 104 donors (33.4 %). Grade I complications occurred in 71 (57.7%), grade II complications in 9 (7.3%), grade IIIa in 39 (31.7%), grade IIIb in 3 (2.5%), and grade V complication in 1 patient (0.8%). The rate of rehospitalization was about 16% in all donors according to various indications (Clavien grades II, III, and V). The incidence of reoperation was 0.9% in all donors (Clavien grade IIIb).

## DISCUSSION

Donor safety is the key issue for the wider application of LDLT. No effort should be spared in avoiding complications by appropriate patient selection, controlling blood loss, meticulous surgical technique, and postoperative care. This would minimize complications and ensure early return to the normal activities of life. LDLT was first applied to pediatric patients using the left lateral segment or left-lobe graft for donor safety. However, after experiencing liver failure in small-sized grafts in adults and large children, it was concluded that the risk-to-benefit ratio would favor the use of right-lobe graft in LDLT.^15^

Right lobectomy from healthy donors was introduced to the Department of Transplantation Immunology, Kyoto University, Kyoto, Japan in February 1998. This has enabled us to minimize postoperative failure in adult patients.^16^ Analysis of postoperative complications in these donors is important because any complication could lead to death.

Since the initiation of the program of right-lobe donation until the end of December 2003, 311 hepatic right liver transplants were performed. Postoperative analysis of complications revealed the occurrence of 123 complications in 104 donors (33.4% of donors). The incidence and the severity of complications were found to decrease by acquiring more experience. This was true with regard to technical problems (vascular and biliary). One donor developed portal vein thrombosis which occurred early in the protocol. Biliary problems occurred in 12% of the donors, which also decreased by the surgical teams' gaining experience in the technique of harvesting. Biliary complications had occurred in 22 of the first set of 155 donors (14%) and in 15 of the second set of 156 donors (9.5%). This improvement indicates the importance of the learning curve.

The incidence and variety of complications in living donors vary with the program and with the donor population. The differences are also affected by the lack of uniformity in assessing and defining complications. Published reports on donor outcomes indicated a wide range of complication rates that varied between 9 and 67%.^4^,^17^–^23^ This may be attributed to the change in the definition of each complication.

In the present study, biliary complications constituted 40 episodes in 37 donors (about 12%). Bile leakage including minor and self limiting leakage occurred in 34 donors (about 11%). The cause of the leakage was the cut surface of the liver in 30 donors (81%) and from the bile duct in three donors (8%) but not defined in four donors (11%). Biliary stricture occurred in six donors (about 2%); three of the six cases (2%) of biliary strictures followed bile leakage. One donor with stricture required conversion to Roux-en-Y hepaticojejunostomy; cholestasis occurred in 17 donors (5%). Persistent fluid collection is the second most frequent complication and constituted 38 episodes in 38 donors. The rate of rehospitalization was about 16% for all donors according to various indications (Clavien grades II, III, and V). The incidence of reoperation was 0.9% for all donors (Clavien grade III-b).

The incidence of biliary leakage in our series was comparable with that in other studies.^24^ Cases of biliary leakage from the bile duct were followed by stricture which was managed successfully with ERCP. Although great care was adopted in harvesting the donor graft and in cutting the ducts in the donor side with the use of cavitational ultrasonic surgical aspirator (CUSA) and clipping and tying the small bile ducts on the cut surface, the resolution of biliary complications was still slow. This may be due to the selection of donors with more than one bile duct or with complex biliary anatomy as the only available donors for critically ill recipients. Thus, our learning experience must have clearly decreased the incidence of biliary complications although the improvement was not statistically apparent due to the increase in acceptance of donors with complex biliary anatomy. In other series, approximately 40% of right-lobe donors experienced at least one complication during the year after the transplant surgery.^24^ Biliary leakage or stricture complicated the postoperative course in 6% of the donors,^25^ an observation confirmed by previous reports.^26^,^27^ Rehospitalization occurred in 8.5% of 75 donors, 5% of whom required reoperation.^25^ A recent multicenter survey in five Asian liver transplantation centers reported complications and long-term outcomes in 1,058 live donors: more than half of these were right-lobe donors (561 of 1,058 donors).^28^ Although the overall morbidity rate was 15.8%, right-lobe donors had a greater incidence of complications (28%) and more serious complications compared with left-lateral segment (9%) and left-lobe donors (7.5%) respectively. The most common serious problem encountered in patients undergoing right-lobe donation was biliary complications, in particular, cholestasis, (7%), bile leakage (6%), and biliary stricture (1%). Other serious complications included portal vein thrombosis (0.5%), intra-abdominal bleeding (0.5%), and pulmonary embolus (0.5%). Reoperation was necessary in 17 donors (1.1%) for small bowel obstruction, biliary stricture, bile leakage, bleeding, portal vein thrombosis, postoperative ileus, and incisional hernia. Only 15% of patients were followed up for longer than three months; however, six donors had residual morbidity, including five patients with biliary complications and one patient with chronic renal failure secondary to intravenous contrast. One donor experienced sudden death while exercising three years after the transplant.^28^

A review of the experience in the USA with adult LDLT reported a 4% biliary complication rate in donors who required surgical intervention, endoscopic retrograde cholangiopancreatography, or percautaneous catheter drainage of a postoperative biloma.^29^ Surgical re-exploration was required in two patients for portal vein thrombosis and small-bowel obstruction. Additional complications reported in the donor population include neuropraxia, phlebitis, pressure sores, pleural effusion, pneumonia, pulmonary embolus, deep venous thrombosis, prolonged ilieus, and incisional hernia.^29^

Beavers et al. reviewed the incidence of donor morbidity associated with right lobectomy in LDLT between 1995 and 2001.^30^ Reported complications in right-lobe donors ranged from 0 to 67% with a crude complication rate calculated to be 31% (54 events in a 74 donors). The wide variation in complication rates among centers reflects the inconsistency in defining what constitutes a complication, with some centers reporting all adverse events and others reporting only major or life-threatening complications. On an average, right-lobe donors spent 9.9 days in the hospital, returned to work 2.4 months postoperatively, and felt completely recovered by 3.4 months.^30^

There was an acceptably good correlation between estimated graft volume and actual graft weight, as reported previously.^10^ However, a significant error was observed in some cases, which may have contributed to smaller estimated RLV than the actual one, or may have been attributable to an intentional shift of the transaction plane to the left side on the basis of sufficient size of the estimated RLV.

One donor died of liver failure due to a small remnant liver (26%) and concomitant nonalcoholic steatohepatitis that was diagnosed postoperatively, in spite of rescue domino transplant. This is the first donor death to be reported from Asia; the donor was a woman who donated her liver to her teenage daughter.^31^ The preoperative assessment showed mild to moderate steatosis and the residual volume was estimated to be 30%. The actual volume after the operation was 26% which may have been due to the shift of the resection margin to the left. The lesson learned from this case was the need to avoid hesitation in taking liver biopsy from donors, especially when the estimated residual volume was ≤30%, so that no additional risk factors, such as steatohepatitis, were imposed on the remaining part of the liver A total of nine deaths occurred worldwide (two deaths in left lateral segmentectomy donors and seven deaths in right lobectomy donors). Three additional donors have undergone liver transplantation because of complications related to right-lobe donation.^28^,^31^,^32^ Unlike the two deaths of left lateral segmentectomy donors (pulmonary embolus and anaphylaxis), deaths occurring in right-lobe living donors have resulted from multiple organ failure and sepsis. Although donor deaths have resulted from either technical failures or problems in the early postoperative period, these cases underscore the reality that living donation is associated with a small, but real, possibility of mortality. The increasing use of living-related donation should not delay the seeking of ways to obviate the need for transplantation. Also, the optimal graft is a whole liver from a cadaveric donor. Our efforts must go into making full use of the cadaveric pool. We must know that only a proportion of candidates will be helped by living liver donors either due to scanty or due to medical contraindications in living donors.

Finally, the morbidity associated with right-lobe donation is significant and probably occurs in a third of all the donors. However, close attention and meticulous techniques must be used to prevent surgical complications such as bile leakage and cholestasis. Vigilant examination is required to avoid inappropriate donor selection. Liver biopsy can be resorted to in donors with a suspicious steatosis.

In the present study, donors were continuously followed up for any complication. The precise rate of incidence of late complications in right-lobe donors is speculative because few, if any, studies exist reporting the follow-up of donors beyond the first postoperative year. Fortunately, all the donors' liver function test results normalize by the end of the first month after liver donation and before regeneration is complete.

In conclusion, LDLT using right lobe grafts for larger patients is another step that can increase donor risk. Donor safety has been and must continue to be the primary measure of success of this technique. With technical refinements backed with precise knowledge on anatomical variations and physiology, we believe right lobe LDLT has become a very effective treatment modality with acceptable risk to both donors and recipients.
